# Temporal and Spatial Blood Feeding Patterns of Urban Mosquitoes in the San Juan Metropolitan Area, Puerto Rico

**DOI:** 10.3390/insects12020129

**Published:** 2021-02-02

**Authors:** Matthew W. Hopken, Limarie J. Reyes-Torres, Nicole Scavo, Antoinette J. Piaggio, Zaid Abdo, Daniel Taylor, James Pierce, Donald A. Yee

**Affiliations:** 1 Department of Microbiology, Immunology, and Pathology, Colorado State University, Fort Collins, CO 80523, USA; Zaid.Abdo@colostate.edu (Z.A.); pierce4193@gmail.com (J.P.); 2 United States Department of Agriculture National Wildlife Research Center, Fort Collins, CO 80521, USA; toni.j.piaggio@usda.gov (A.J.P.); Daniel.Taylor3@usda.gov (D.T.); 3 School of Biological, Environmental, & Earth Sciences, University of Southern Mississippi, Hattiesburg, MS 39406, USA; Limarie.ReyesTorres@usm.edu (L.J.R.-T.); nicole.a.scavo@gmail.com (N.S.); donald.yee@usm.edu (D.A.Y.)

**Keywords:** *Aedes aegypti*, bird, *Culex quinquefasciatus*, mammal, metabarcoding, iDNA, species diversity, vector-borne diseases, high-throughput sequencing

## Abstract

**Simple Summary:**

Understanding the biodiversity of urban ecosystems is critical for management of invasive and pest species, conserving native species, and disease control. Mosquitoes (Culicidae) are ubiquitous and abundant in urban ecosystems, and rely on blood meals taken from vertebrates. We used DNA from freshly blood-fed mosquitoes to characterize the diversity of vertebrate host species in the San Juan Metropolitan Area, Puerto Rico. We collected two mosquito species that fed on a variety of vertebrates. *Culex quinquefasciatus* fed on 17 avian taxa (81.2% of blood meals), seven mammalian taxa (17.9%), and one reptilian taxon (0.85%). *Aedes aegypti* blood meals were from a less diverse group, with two avian taxa (11.1%) and three mammalian taxa (88.9%) identified. Domestic chickens dominated the blood meals of *Cx. quinquefasciatus*, both temporally and spatially, and no statistically significant shift from birds to mammals was detected. The species we detected from the mosquito blood meals provided a snapshot of the vertebrate community in the San Juan Metropolitan Area, most of which were domestic species. However, we also identified a variety of native and nonnative wild species. These results add knowledge about potential ecological factors that impact vector-borne disease management in urban habitats.

**Abstract:**

Urban ecosystems are a patchwork of habitats that host a broad diversity of animal species. Insects comprise a large portion of urban biodiversity which includes many pest species, including those that transmit pathogens. Mosquitoes (Diptera: Culicidae) inhabit urban environments and rely on sympatric vertebrate species to complete their life cycles, and in this process transmit pathogens to animals and humans. Given that mosquitoes feed upon vertebrates, they can also act as efficient samplers that facilitate detection of vertebrate species that utilize urban ecosystems. In this study, we analyzed DNA extracted from mosquito blood meals collected temporally in multiple neighborhoods of the San Juan Metropolitan Area, Puerto Rico to evaluate the presence of vertebrate fauna. DNA was collected from 604 individual mosquitoes that represented two common urban species, *Culex quinquefasciatus* (*n* = 586) and *Aedes aegypti* (*n* = 18). *Culex quinquefasciatus* fed on 17 avian taxa (81.2% of blood meals), seven mammalian taxa (17.9%), and one reptilian taxon (0.85%). Domestic chickens dominated these blood meals both temporally and spatially, and no statistically significant shift from birds to mammals was detected. *Aedes aegypti* blood meals were from a less diverse group, with two avian taxa (11.1%) and three mammalian taxa (88.9%) identified. The blood meals we identified provided a snapshot of the vertebrate community in the San Juan Metropolitan Area and have potential implications for vector-borne pathogen transmission.

## 1. Introduction

Urban environments contain complex ecosystems encompassing a patchwork of different habitats where humans cohabitate with many wild and domestic species. This habitat complexity is dynamic and can harbor high levels of biodiversity [1]. Insects are highly abundant within cities, and some species have adapted specifically to human-modified habitats (e.g., *Aedes* (*Stegomyia*) *aegypti* (Linnaeus)) [2]. Given that insects are widely distributed in cities, represent all trophic levels, are easily collected, are intricately linked to plant and vertebrate diversity, and rapidly respond to habitat alterations, elucidating their ecology can provide insights into urban biodiversity and pathogen transmission [3].

Mosquitoes (Diptera: Culicidae) are ubiquitous and broadly distributed across urban ecosystems [4]. However, mosquitoes are not as well studied in urban areas compared to other insects, with only a few studies focusing on their diversity patterns [5,6,7]. As the density and abundance of mosquitoes are inextricably linked to habitat diversity and plant and vertebrate species as pollinators, food sources, and ectoparasites, urban habitats can determine mosquito species’ presence and abundance, and potential pathogen transmission [8]. For example, using blood meal DNA to detect vertebrate species may help understand vertebrate diversity within the urban network, pathogen transmission pathways, and vertebrate ranges across urban–rural gradients. Previous studies have suggested that some mosquito species’ host selection in urban areas is neighborhood-specific and related to vertebrate host abundance and socioeconomic status [9].

Biodiversity surveys are a major component of ecology, conservation biology, and epidemiology. There are multiple field-data collection methods useful for the quantification of biodiversity, and with increasing technological advances, more non-traditional methods are available to researchers. Genomic technology is one such method that has played an increasing role in biodiversity sampling [10]. Environmental DNA (eDNA), which is DNA shed into the environment and collected without handling the organism, has allowed researchers to survey and detect a broad array of species, which has improved ecological studies, invasive species management, disease mitigation, and conservation [11,12]. An extension of the eDNA concept is the use of DNA from hematophagous invertebrates, called invertebrate-derived DNA (iDNA) [13], to detect vertebrate species in an ecosystem. Invertebrate-derived DNA has been used successfully in biodiversity surveys and the detection of rare, elusive species [14,15]. Through the collection of iDNA, we gain knowledge about invertebrate biology, such as how host presence affects invertebrate distribution, and document temporal and spatial variation in community composition.

DNA metabarcoding using high-throughput sequencing (HTS) has become a common practice in biodiversity and eDNA studies [16,17]. Using this approach, researchers can sequence DNA from multiple species in a mixed-species sample (e.g., water, soil, feces), and estimate biodiversity for inferences about ecological community composition, changes of communities in response to a disturbance, and determining the major components of an organism’s diet [18,19]. However, to date this approach has been less commonly applied to hematophagous insect blood meal identification [20]. One main advantage of HTS over methods like Sanger sequencing is that all molecules in a sample can be sequenced simultaneously, which allows for less-biased species identification and detection.

Using iDNA to identify host selection and to evaluate diet specialization of insects is not a new concept. This approach has been used for decades in vector-borne disease ecology [21]. Identifying vector and host interactions aids discovery of potential vector species, susceptible hosts, potential reservoir species, and facilitating studies of host-parasite-vector co-evolution [21]. In addition, quantification of hematophagous invertebrate host diversity helps determine vector biting rates on susceptible hosts, which is a critical parameter in the vectorial capacity model [22,23]. Goodman et al. [9] demonstrated that mosquito blood meals in urban environments can directly reflect the abundance of hosts. They found that the majority of mosquito blood meals from an urban neighborhood in Baltimore, Maryland, USA, were identified as brown rat (*Rattus norvegicus* (Berkenhout), which correlated with very high densities of this species.

In this study, we utilized samples collected as part of a complementary mosquito biodiversity study in the San Juan Metropolitan Area, Puerto Rico, USA (Scavo et al. in revision) to evaluate if host selection varies spatially or temporally across different neighborhoods based on socioeconomic status (SES). Both DNA metabarcoding and Sanger sequencing were used to analyze iDNA from freshly engorged specimens of *Culex quinquefasciatus* (Say) and *Ae. aegypti* collected in multiple neighborhoods over 16 months. We identified a diversity of hosts that included humans as well as wild and domestic vertebrate species. The results from this study provide insight into mosquito host selection in a subtropical, urban environment and demonstrate the utility of blood meal metabarcoding as a tool to detect urban vertebrate biodiversity and identify potential pathogen transmission pathways.

## 2. Materials and Methods

### 2.1. Study Site

Puerto Rico is a Caribbean archipelago that has an area of approximately 9104 km², and a population of over three million people (U.S. Census Bureau, 2018). San Juan (18°27′ N, 66°05′ W), the capital, has a population of approximately 350,000 (U.S. Census Bureau, 2018). The San Juan Metropolitan Area (SJMA) has a subtropical, maritime climate, with the rainy season occurring from May to October. Elevation increases and level of urbanization decreases moving south from the city center. The municipalities that make up the SJMA present a mosaic of highly urbanized areas, wetlands, urban forest fragments, and managed green spaces [24]. There is limited knowledge of mosquito species distribution within the SJMA [5], and even less knowledge about host selection in the urban environment.

**Figure 1:**
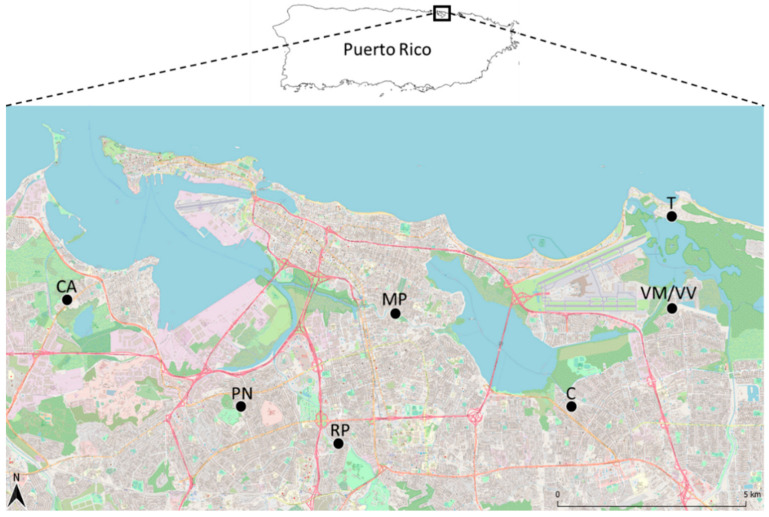
Map of mosquito-trapping locations in the San Juan Metropolitan Area, Puerto Rico in 2018 and 2019. The inset highlights the location of San Juan on the island of Puerto Rico. Each of the black dots represent the centroid of latitude and longitude of all traps deployed in the neighborhood. The neighborhood abbreviations are: CA—Cataño, MP—Martin Peña, PN—Puerto Nuevo, RP—Río Piedras, T—Torrecilla, VM—Vistamar, VV—Villa Venecia. VM and VV were combined into a single point on the map because of their geographic proximity.

Sampling occurred in eight neighborhoods across three municipalities (San Juan, Carolina, and Cataño) (Figure 1). Neighborhoods were chosen to represent a gradient of socioeconomic and ecological factors (Table 1). Two neighborhoods in proximity, Villa Venecia and Vistamar, have similar surrounding habitat but different SES (former neighborhood is high SES and gated). Torrecilla is surrounded by mangrove forests and saltwater habitats. Cataño and Martin Peña are characterized by closely placed housing and semi-frequent flooding from canals within the communities. Puerto Nuevo is near a large, forested park.

### 2.2. Human and Landscape Variables

Neighborhood heterogeneity was quantified using human (SES) and landscape variables. Socioeconomic status was based on U.S. Census data (2018) (Table 1). Park size and amount of litter/trash were included since they have been shown to affect mosquito abundance and diversity [25,26]. Additionally, distance to the nearest water body also affects mosquito community composition [27]. Water body presence was included due to its role as a mosquito habitat and as a proxy for likelihood of flooding. Foot surveys (*n* = 103) were conducted along 50 m transects to assess levels of abandonment, type of spaces (e.g., water body, park), and incidence of litter, the latter of which can provide rearing sites for aquatic mosquito larvae.

### 2.3. Mosquito Adult Sampling and Sample Preservation

Samples were taken in January, March, May, and October of 2018, and in January and May of 2019. Adult mosquitoes were collected using BG Sentinel 2 traps (Biogents, Regensburg, Germany) baited with scented BG lures (Biogents, Regensburg, Germany) set out for 48 h. These traps are designed to attract anthropophilic mosquitoes, especially host-seeking females. During each sampling event, six traps per neighborhood (*n* = 144) were placed outside of residences at least 200 m apart from each other.

CDC light traps (Bioquip, Rancho Dominguez, CA, USA) baited with CO_2_ were used in tandem with BG Sentinel 2 traps in four residences per neighborhood (*n* = 96). These traps were used to sample the wider mosquito community, as they are designed to attract any insect that cues on light. At residences with both trap types, traps were placed at least 10 m apart to avoid competition between traps. Small coolers (Igloo thermos, ½ gal.) filled with dry ice were placed next to light traps with a plastic tube directing the released CO_2_ toward the entrance of the trap. The addition of CO_2_ would increase the likelihood of attracting host-seeking female mosquitoes. Light traps were hung at approximately 1.5 m high and left for 48 h. Mosquitoes from traps were frozen at −20 °C and later identified to genus (for *Culex*) and species (for *Ae. aegypti*) using *The Mosquitoes of Puerto Rico* (Tulloch, 1937) and *Key to the Mosquito Genera of Puerto Rico* (Barrera, unpublished) based on morphological characters. Blood-engorged females were identified based on the visual inspection of a distended abdomen and dark coloring between the abdominal sclerites, and were preserved in 95% ethanol for subsequent analysis.

### 2.4. DNA Extraction

Blood-engorged abdomens were removed from female mosquitos using forceps and a microprobe, which were flame-sterilized between samples. We extracted genomic DNA from the abdomens using the DNeasy blood and tissue kit (Qiagen, Hilden, Germany) according to Hopken et al. [28]. To limit human contamination of samples, dissections and extractions were performed in a biosafety cabinet located in the Wildlife Genetics Laboratory of the USDA-APHIS National Wildlife Research Center, Fort Collins, Colorado, USA in a room dedicated to processing eDNA and non-invasive samples. The head and thorax from each specimen were placed in absolute ethanol and stored at −20 °C in case morphological identification needed to be revisited. Each extraction included a blank containing only reagents and processed with each batch of samples to monitor for contamination.

### 2.5. Molecular Verification of Mosquito Species

We randomly selected 10% of the individuals morphologically identified as *Cx. quinquefasciatus* from each trapping site for molecular verification of species identification. We sequenced approximately 650 base pairs (bp) of the mitochondrial cytochrome oxidase subunit I (COI) gene following Hopken et al. [28] using primers BFculicFm1 [29] and C1-N-2191 [30]. Sanger sequencing was conducted on a Genetic Analyzer 3500xl (Thermo Fisher Scientific, Waltham, MA, USA). We assembled and edited forward and reverse sequences using Sequencher^®^ version 5.4.6 (Gene Codes Corporation, Ann Arbor, MI, USA) and compared to references available in the NCBI Genbank using the Basic Local Alignment Search Tool (BLAST; https://blast.ncbi.nlm.nih.gov/Blast.cgi) [31]. Species identification was based on 98% sequence identity [32].

### 2.6. DNA Metabarcoding

We employed a two-step PCR amplicon sequencing approach targeting around 110 bp of the mitochondrial small subunit ribosomal RNA (12S) gene to identify vertebrate species from blood meal DNA extracts [18]. We used the 12S PCR primers 12SV5F/12SV5R from Riaz et al. [33] and added heterogeneity spacers to account for low-diversity libraries and improve sequencing quality. Illumina sequencing primer sequences were added to the 5′ end of the amplification primers, which acts as a binding site for the primers in the second PCR, which included the sequencing primer, indexes to identify individual species, and Illumina sequencing adaptors (Illumina, San Diego, CA, USA; Supplementary Table S1).

The first PCR contained a mix of four forward primers and four reverse primers, each with the 12SV5 primer sequence, one of four heterogeneity spacers, and a partial sequencing primer sequence. The first-round PCR volume for each sample was 15 µL and contained 7.5 µL of Qiagen 2X multiplex PCR master mix, 4.5 µL DEPC-treated H_2_O, 0.5 µL of each primer mix at 10 µM, and 2 µL of DNA extract. Thermocycling conditions were an initial denaturation at 95 °C for 15 min (min), followed by 35 cycles of 94 °C for 30 s (sec), annealing at 45 °C for 1 min 30 s, extension at 72 °C for 2 min, and a final extension at 72 °C for 10 min. Amplified PCR products were purified with Mag-Bind^®^ TotalPure NGS beads (Omega Bio-tek, Inc., Norcross, GA, USA) following the manufacturer’s protocol modified to 1.4 volume of beads and 50 µL elution in Qiagen buffer EB. Purification steps were automated using an Opentrons OT-2 liquid handling robot (Opentrons, Brooklyn, NY, USA).

Dual indexes and sequencing adaptors were added to the purified products in a second PCR. The 15-µL PCR volume for each sample contained 7.5 µL of Qiagen 2X multiplex PCR master mix, 2.9 µL DEPC-treated H_2_O, 1.8 µL of each primer at 10 µM, and 1 µL of purified product from the first PCR after diluting 1:1 in DEPC-treated H_2_O. Thermocycling conditions were an initial denaturation at 95 °C for 15 min, followed by 8 cycles of 95 °C for 15 s, annealing at 55 °C for 45 s, extension at 72 °C for 1 min, and a final extension at 72 °C for 10 min. Amplified PCR products were purified with Mag-Bind^®^ TotalPure NGS beads (Omega Bio-tek, Inc., Norcross, GA, USA) again with 1.4 volume of beads and 50 µL elution. Negative controls (reagents only) were included in each extraction, and no template controls were included in each PCR.

All purified samples were analyzed on a QIAxcel DNA High Resolution Gel Cartridge (Qiagen) to record fragment length of the libraries. We recorded concentration of each sample using a Qubit™ dsDNA HS Assay Kit (Invitrogen, Carlsbad, CA, USA). Samples were then pooled in equimolar volumes and prepared for paired-end sequencing on an Illumina MiSeq System using the 500-cycle MiSeq Reagent Kit v2 (Illumina). We ran the samples on two separate sequencing runs.

### 2.7. Vertebrate Species Verification of Inconclusive Samples

Mosquito blood meals with inconclusive species identification based on 12S amplicon sequencing were verified by Sanger sequencing a 5′ portion of the COI gene using primers from Townzen et al. [34]. We first attempted to amplify the COI gene using the primers COI_long f/r. If a sample failed, we then attempted to amplify a shorter fragment of the same COI region using primers COI_short f/r following Pettersson et al. [35].

The 25 µL PCR to amplify COI_long contained 15.35 µL DEPC-treated H_2_O, 2.5 µL 10× buffer II (Thermo Fisher Scientific), 1.5 µL of 25 mM MgCl_2_, 1.25 µL of dNTPs at 2.5 mM, 1 µL of each primer at 10 µM, 0.4 µL of Amplitaq Gold polymerase (Thermo Fisher Scientific), and 2 µL of DNA extract. Thermocycling conditions to amplify COI_long were an initial denaturation at 95 °C for 15 min, followed by 15 cycles at 95 °C for 40 s, a touchdown annealing temperature of 55 °C for 45 s that decreased 0.5 °C each cycle to 47.5 °C, extension at 72 °C for 1 min, followed by 32 cycles at 95 °C for 40 s, annealing at 45 °C for 40 s, extension at 72 °C for 45 s, and a final extension at 72 °C for 7 min.

The 25 µL PCR to amplify COI_short contained 16.1 µL DEPC-treated H_2_O, 2.5 µL 10× buffer II (Thermo Fisher Scientific), 1 µL of 25 mM MgCl_2_, 1 µL of dNTPs at 2.5 mM, 1 µL of each primer at 10 µM, 0.4 µL of Amplitaq Gold polymerase (Thermo Fisher Scientific), and 2 µL of DNA extract. Thermocycling conditions to amplify COI_short were an initial denaturation at 95 °C for 15 min, followed by 45 cycles of 94 °C for 30 s, annealing at 50 °C for 45 S, extension at 72 °C for 30 s, and a final extension at 72 °C for 10 min.

Amplified products were visualized on a QIAxcel DNA High Resolution Gel Cartridge (Qiagen). We removed unincorporated primers and dNTPs from PCR product using an enzymatic digestion (ExoSAP-IT^®^, Affymetrix Inc., Santa Clara, CA, USA). Cycle sequencing was performed in both directions using a BigDye^®^ Terminator V3.1 kit (Applied Biosystems, Foster City, CA, USA), and cycle sequencing products were purified with a Sephadex clean-up using 96-well filter plates (Whatman, Maidstone, UK). Purified products were denatured and Sanger sequenced on an Applied Biosystems 3500xl genetic analyzer. Forward and reverse sequences were assembled and edited using Sequencher^®^ version 5.4.6 (Gene Codes Corporation, Ann Arbor, MI, USA) and compared to references available in Genbank using the Basic Local Alignment Search Tool (BLAST; Altschul et al. 1990). Species identification was based on 98% sequence identity.

### 2.8. Bioinformatics and Statistical Analysis

Illumina sequencing reads were quality filtered and trimmed using trimmomatic v0.36 [36]. Sequence read processing was completed with various commands of usearch v11.0.667 [37], unless cited otherwise, and command settings are provided in Supplementary Table S2 Paired reads were merged with usearch -fastq_mergepairs. An additional quality filtering step of the merged pairs was conducted with vsearch v2.13.3 command -fastq_filter [38]. We dereplicated the merged sequence reads using usearch -fastx_uniques and clustered operational taxonomic units (OTUs) at the 97% sequence identity level using usearch -cluster_otus. OTUs were annotated with sequence read depth using usearch -otutab.

We assigned taxonomy to the OTUs using two approaches. The first approach was with usearch -usearch_global command and the MIDORI reference data base, which includes mtDNA sequences from Eukaryotic organisms (reference-midori.info/index.html). We downloaded all the 12S (small subunit ribosomal DNA) sequences in SINTAX format. The database was downloaded on 16 October 2020. The second approach was to use blast to compare the OTUs against the NCBI database using the command blastn [31]. The searches were conducted on 22 October 2020. For all species identifications, we used a 98% identity as a threshold, and for any OTU identifications that were below this match, we followed up with Sanger sequencing of the COI locus. If this did not improve species identification, we reported the organism at its lowest taxonomic level possible (meaning that the hits in NCBI or MIDORI all matched to the same genus, family, etc.).

**Figure 2:**
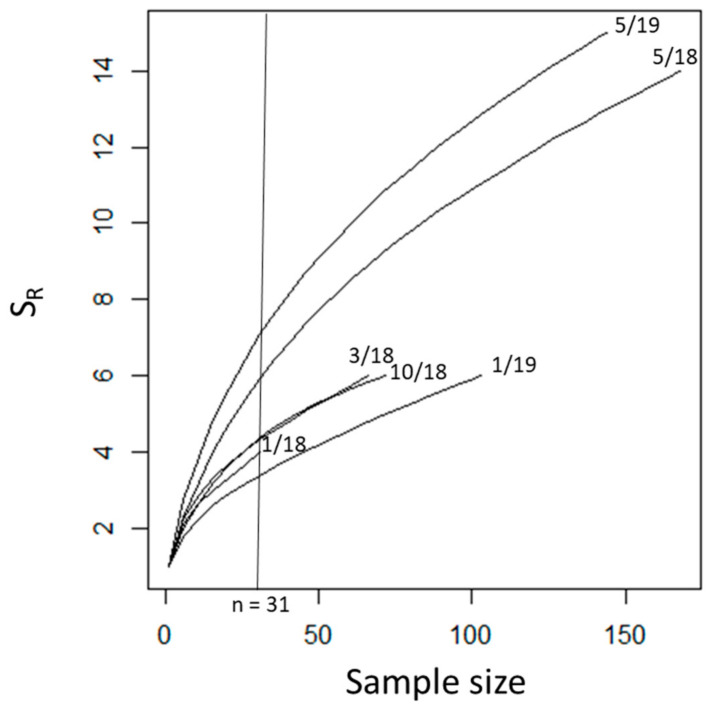
Rarefaction curves for vertebrate species identified from mosquito blood meals collected at six trapping dates in the San Juan Metropolitan Area, Puerto Rico. The x-axis is sample sizes, the y-axis is species richness (*S_R_*), and the vertical line represents the small sample size, which was used for rarefaction. The tips of each curve are labeled with the trapping date in the month/year format.

Given that our sample sizes of *Ae. aegypti* were small, we conducted all statistical analyses on *Cx*. *quinquefasciatus* blood meals only. We used a rarefaction curve produced by the vegan package in R v4.0.2 to evaluate the relationship between host-species richness and sample sizes (Figure 2). We calculated Shannon entropy (*H′*), Gini–Simpson (*D*), and rarefied species richness (*S_R_*, normalized to *n* = 31 for the temporal sampling only) using the vegan package. *Culex quinquefasciatus* is an opportunistic feeder that prefers avian hosts. However, it will switch to mammals under certain environmental conditions. Accordingly, we tested for differences in species counts per trapping date, and per trapping site using a Fisher’s exact test. We also compared the number of avian versus mammalian-derived blood meals across trapping dates using a Fisher’s exact test. Pairwise Bray–Curtis distances between trapping dates were calculated and clustered with vegan, then a dendrogram was plotted in R.

## 3. Results

### 3.1. Blood-Engorged Mosquito Collection and Identification

**Figure 3:**
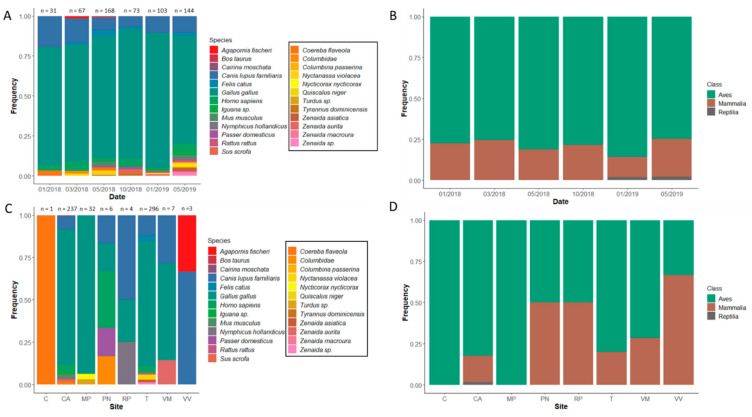
Barplots of taxonomic identification of DNA sequences from *Culex quinquefasciatus* collected in the San Juan Metropolitan Area, Puerto Rico. Plots (**A**) and (**B**) are divided by trapping date. Plots (**C**) and (**D**) are divided by trapping site. The x-axis provides either the trapping date or trapping site, and the y-axis is the frequency of taxon detection. The colors within the plot represent different vertebrate species (plots (**A**) and (**C**)) or vertebrate classes (plots (**B**) and (**D**)). Sample sizes per division are provided above the bars in plots (**A**) and (**C**). The black squares around the taxa in the legends in plots (**A**) and (**C**) are native wildlife, while the non-enclosed taxa are human commensals/introductions.

We obtained a total of 698 blood-engorged individuals for sequencing, with successful vertebrate species identification for 604 (86.5%) blood meals (Table 2, Table S3). Sample sizes per trapping date ranged from 31–168 (Table 3, Figure 2A) and per site ranged from 1 to 296 (Table 4, Figure 3C). Most of the successful samples were from *Cx. quinquefasciatus* (*n* = 586; 97%) and the remaining were *Ae. aegypti* (*n* = 18; 3.0%). Sanger sequencing of the COI gene of 46 randomly selected mosquito individuals confirmed the morphological identification. The initial BLAST results returned a >98% match to either *Cx. quinquefasciatus* or *Cx. pipiens* L (every specimen had identical matches to both species) for all except two specimens. However, only *Cx. quinquefasciatus* has been recorded in Puerto Rico, so we considered this match to be confirmation of species identification to *Cx. quinquefasciatus*. Of the two individuals that did not match >98% to *Cx. quinquefasciatus*, one individual was maximum match at 97.2% and the other individual was a >99% match to both *Cx. nigripalpus* Theobald and *Cx. conspirator* Dyar and Knab, with *Cx. nigripalpus* being most likely in Puerto Rico. DNA sequences for the mosquito species were submitted to NCBI GenBank under accession numbers MW509569-MW509611.

### 3.2. Molecular Blood Meal Identification

The two MiSeq runs combined resulted in 27.63 million paired-end reads. Following quality filtering and full data processing, the average sequence depth was 2895.04 per sample (range: 137–22865; Supplementary Table S3).

The sequences resulting from 12S amplicon sequencing matched to a host species at >98% sequence identity for 560 (92.7%) samples. All of the low-confidence matches were blood meals from *Cx. quinquefasciatus*. Sanger sequencing of COI refined the species identification in 30 (68.1%) of the 44 unidentified samples. Combining the results from 12S amplicon and COI Sanger sequencing for both mosquito species provided 594 (98.3% of 604 taxon identifications) species-level identifications. The remaining 10 samples were at least identified to family, with nine identified to genus (Table 2). Only one negative control (an extraction blank) returned sequence data with a depth of 306, and the BLAST search returned *Homo sapiens*. Our human blood meal identifications were at much higher sequencing depths, thus we feel comfortable considering the reads in the extraction blank as background contamination. We did encounter a low level of human DNA in some of the samples that were identified as other species, but we were able to discard these OTUs, as they were below the 10% filtering threshold. All 12S and COI sequences were submitted to NCBI GenBank under accession numbers COI: MW464127-MW464167 and 12S: MW524152-MW524744. Raw sequence reads were submitted to the NCBI Sequence Read Archive under accession number PRJNA697970.

*Culex quinquefasciatus* fed upon a total of 25 taxa across our trapping sessions. Birds made up the largest proportion of *Cx. quinquefasciatus* blood meals (*n* = 476; 81.2%; Table 2). Birds also dominated the taxonomic diversity, with 17 (68%) taxa versus 7 (28%) mammals and 1 (4%) reptile. A single species, chicken (*Gallus* (Linnaeus)), accounted for most of the *Cx. quinquefasciatus* host identifications (*n* = 443; 75.6%). The largest diversity of blood meals was from wild birds, with 12 taxa considered wild native birds, and 1 taxon was a wild non-native bird (house sparrow (*Passer domesticus* (Linnaeus)); *n* = 1; Table 2, Figure 2). The remaining bird taxa consisted of two pet species, cockatiel (*Nymphicus hollandicus* (Kerr); *n* = 1) and Fisher’s love bird (*Agapornis fischeri* Reichenow; *n* = 1), and a domestic Muscovy duck (*Cairina moschata* (Linnaeus); *n* = 1). Five avian blood meals could not be identified to species, but three were identified to genus as doves (*Zenaida* sp.). There are multiple wild species in Puerto Rico that belong to this genus (we identified three species from blood meals), thus we cannot make assumptions about species identity based on distribution. The other two avian blood meals without species identifications belonged to the Columbidae family (doves and pigeons) and the *Turdus* genus (thrushes). We also identified several wild bird species, including two heron species (*Nyctanassa violacea* (Linnaeus), *Nycticorax nycticorax* (Linnaeus)) from five blood meals, four Greater Antillean grackles (*Quiscalus niger* (Boddaert)), three Bananaquits (*Coereba flaveola* (Linnaeus)), and a gray kingbird (*Tyrannus dominicensis* (Gmelin)).

Mammals were identified from 105 (17.9%) *Cx. quinquefasciatus* blood meals (Table 2). The dominant mammal host in our dataset was domestic dog (*Canis lupus familiaris* Linnaeus; *n* = 60; 57% of mammal blood meals), followed by humans (*n* = 20; 19% of mammals; 3.4% of total). The remaining mammal blood meals consisted of domestic cats (*Felis catus* (Linnaeus)), two human commensal rodents (*Mus musculus* (Linnaeus) and *Rattus rattus* (Linnaeus)), and swine (*Sus scrofa* (Linnaeus)).

Six *Cx. quinquefasciatus* mixed blood meals were identified that had multiple species detected that were over the 10% sequence read threshold. The samples were all *Cx. Quinquefasciatus*, and the host species combinations were chicken/domestic cat (*n* = 2), chicken/human (*n* = 2), domestic dog/human (*n* = 1), and domestic dog/chicken (*n* = 1). Overall, multiple blood meals for *Cx. quinquefasciatus* were rare in our samples (6/586 = 1.02%).

We only obtained 18 blood meal identifications from *Ae. aegypti* (Table 2). Sixteen (89%) of the blood meals were from mammals, with the dominant being domestic dog, followed by human and domestic cat. We detected two birds, a chicken and a green heron (*Butorides virescens* (Linnaeus)).

**Figure 4:**
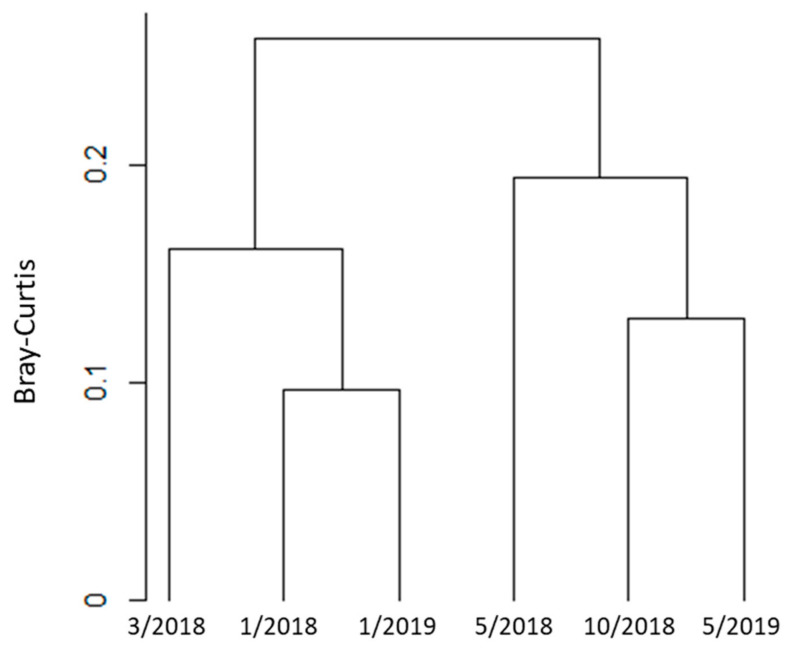
Dendrogram based on cluster analysis of the Bray–Curtis distance. The tips represent six trapping dates (month/year format) in the San Juan Metropolitan Area, Puerto Rico. The y-axis represents the Bray–Curtis distance estimated from vertebrate species diversity identified through mosquito blood meals.

The distribution of *Cx. quinquefasciatus* blood meal identifications across trapping dates and sampling sites was variable (Table 3 and Table 4, Figure 2). Qualitatively, the highest species diversity was in May of both 2018 and 2019 (the start of the wet season). The diversity indices per trapping date were similar (Table 5, Figure 4). The Fisher’s exact test on rarefied species counts per date was not significant (*p* = 0.473). Comparing just the largest sample sizes without rarefaction, May 2018 to May 2019, was also not significant (*p =* 0.116). Finally, comparing avian versus mammalian blood meals across trapping dates was not significant (*p* = 0.279).

Only Cataño and Torrecilla (combined *n* = 533; 91% of all samples) had large sample sizes, and thus we restricted our diversity estimates to these sites. These neighborhoods were two of the lowest SES (Table 1 and Table 4). The per site *H′* for Cataño was 0.874, and for Torrecilla it was = 1.057. The value of *D* for Cataño was 0.355, and for Torrecilla it was 0.441. Non-rarified species richness for Cataño was 11, and for Torrecilla it was 13. Torrecilla had qualitatively higher species richness than Cataño in all indices, and the Fisher’s exact test was significant (*p* < 0.001).

## 4. Discussion

The results of this study demonstrate that iDNA can be a useful tool that complements traditional techniques used by urban ecologists for studying insect host choice and vertebrate biodiversity. We combined HTS and Sanger sequencing to obtain highly confident vertebrate species detections from blood-engorged mosquitoes collected in the SJMA. Vertebrate diversity identified from blood meals revealed limited total diversity, as few species dominated across neighborhoods and sampling time points, and rare detections drove differences in diversity patterns. Although these patterns were not statistically significant, the highest diversity of host species from sampling locations Cataño and Torrecilla during May of 2018 and 2019 coincided with the highest mosquito diversity and sample sizes (Scavo et al. in revision).

We achieved high species identification success rates by combining metabarcoding and Sanger sequencing that mirrored or improved on other studies of blood meal identification, e.g., [21]. This approach allowed us to overcome some of the issues associated with molecular identification of blood meal sources, such as degraded samples and blood meals taken from multiple species. Metabarcoding alone allowed recovery and easy identification of blood meals from multiple hosts without necessitating multiple lab assays. An additional benefit of metabarcoding is that many more samples can be sequenced in a single run when compared to Sanger sequencing, and overall the cost per sample is less [20]. Short-read HTS targets smaller DNA molecules, thus partially degraded samples can still be recovered with high success, resulting in fewer discarded samples [14]. However, short-read sequencing can limit taxonomic resolution for closely related species, which is a problem we encountered for a few specimens (Supplementary Table S3). To overcome these issues, one can target multiple loci, which is becoming standard practice in metabarcoding, for identification of mixed-species samples and diet analyses [39], or follow-up on low-confidence species identification with longer, high-quality sequences from Sanger sequencing, as done in this study. The main limitation of metabarcoding for biodiversity surveys is the lack of representation of species diversity within molecular databases used for taxonomic assignment, which can result in low-confidence species identification. For example, one of our samples was only identifiable to the genus *Butorides* using the short 12S sequences. However, the follow-up Sanger sequencing with COI barcoding revealed the species as a green-backed heron (*Butorides virescens*). Due to either species missing from databases, or limited taxonomic resolution of the COI and 12S loci for certain taxa, we were unable to identify the one sample assigned as the genus *Turdus* to a single species. Aside from the few shortcomings, metabarcoding can streamline sequencing of samples and improve species identification, which reduces effort, time, costs, and discarded samples in studies focused on iDNA.

### 4.1. Temporal and Spatial Host Patterns

Host diversity patterns across sampling time points matched what is known about mosquito emergence in Puerto Rico. Mosquito abundance is correlated with rainfall patterns, and in Puerto Rico there are two wet seasons, approximately occurring in April/May and October, with *Cx. quinquefasciatus* known to be the most abundant in the spring [24]. We detected the broadest diversity of mosquito hosts in May of both 2018 and 2019, with the most native wild species detected during these times. The total number of blood-engorged mosquitoes was driven by just two sampling sites, Cataño and Torrecilla (Figure 1; see below), which points to non-independence of sampling site and seasonal abundance. Once corrected for sampling effort through rarefaction, the diversity metrics did not reveal statistically significant or large qualitative differences across time points. Most likely, the number of blood-engorged mosquitoes and detection of host diversity in May is a function of mosquito abundance during these times (Scavo et al., in revision). Goodman et al. [9] found that the highest number of blood-engorged specimens and host diversity in an urban ecosystem was when the highest number of mosquitoes were captured. Unfortunately, we did not collect enough *Ae. aegypti* to draw any conclusive patterns about seasonal host choice in this species, which is likely attributed to the lower abundance of this species in the trapping locations (Scavo et al,. in revision).

Some studies in temperate climates have found that the *Culex* mosquito’s host choice shifts in different seasons, depending on host availability. Kilpatrick et al. [40] found that fall bird migration drove *Cx. pipiens* in a northeastern USA urban area to shift from birds to mammals, which included increased human feeding. Thiemann et al. [41] found shifts of *Cx. tarsalis* toward mammals in the fall in California, USA. However, another study in the southeastern USA did not find a seasonal pattern to host choice [42]. We did not detect a statistically significant trend of *Cx. quinquefasciatus* shifting between birds and mammals over time. In fact, domestic chickens remained the dominant host throughout trapping sessions. In subtropical and tropical habitats, many domestic animals remain outdoors year-round and are easily accessible to mosquitoes. All of the wild bird species we detected in blood meals were year-round residents in Puerto Rico. We detected very few human blood meals that did not vary seasonally. The iDNA data we collected and the findings from Mackay et al. [42] suggest that *Culex quinquefasciatus* may not need to shift to suboptimal hosts, such as humans and other mammals, in warmer climates where a high diversity of bird species are year-round residents.

### 4.2. Spatial Host Patterns

Only two trapping sites, Cataño and Torrecilla, had sample sizes large enough for us to estimate species diversity without losing the majority of the data through rarefaction. These sites were two of the three neighborhoods with the lowest SES estimates and the highest mosquito species richness in an associated study (Table 1; Scavo et al., in revision). As mentioned above, the large number of blood-engorged individuals compared to other trapping sites is likely a function of the larger total sample size of mosquitoes. The abundance of mosquitoes in these neighborhoods is not likely due to trapping bias, as multiple studies have demonstrated that mosquito abundance in urban areas is highest in low-SES neighborhoods [26,43,44]. The factors that drive increased mosquito numbers in low-SES neighborhoods are greater availability of larval rearing sites, a higher density of abandoned buildings, and more plant overgrowth, which provides adult resting sites or can impede mosquito-control efforts. Torrecilla had higher vertebrate species diversity than Cataño, which may be explained by its proximity to the coast and mangrove forests; urban mangrove forests are known to host higher species diversity [45].

Chickens were the predominant blood meal sources in most of the trapping sites, however, the percent of chickens compared to other hosts was highest in Cataño (79.7%), Martin Peña (93.8%), and Torrecilla (73.6%; Table 3). Certain areas of San Juan have large numbers of free-ranging and caged chickens (for eggs, food, or cockfighting), and throughout the study area, these three neighborhoods had the highest number of chickens present (Reyes-Torres, personal observation), which may explain the high percentage of chicken blood meals. Our data qualitatively demonstrate that when birds are present in high abundance and are year-round residents, *Cx. quinquefasciatus* will choose to feed on birds. We also found that *Cx. quinquefasciatus* will feed on a broad range of bird taxa (Anseriformes, Passeriformes, Columbiformes, Galliformes, Pelecaniformes, and Psittaciformes) that are both native and introduced.

### 4.3. General Blood Meal Findings

Two species that we detected demonstrate the power of iDNA to detect rare occurrences (Fischer’s lovebird (*Agapornis fischeri*) and a cockatiel (*Nymphicus hollandicus*)). These species are widely distributed around the globe as pets. However, feral individuals of both of these species have been observed in Puerto Rico, although they are rare sightings, and no breeding populations have been detected [46]. Despite the dominance of chickens and the abundance of native bird species, we were still able to detect these rare feeding events on pet birds. This bolsters the idea that pets, especially ones kept outdoors, may be a component of the blood meals of some urban mosquito species compared to suburban locations [47].

Besides birds, we found that *Cx. quinquefasciatus* sometimes fed on urban mammals. The second most abundant blood meal in our data set was from domestic dogs. In San Juan, dogs can be pets, but there are also feral dogs roaming the city, so the results were not surprising [48]. The other non-human mammals that we detected were all human commensals, whether kept for agriculture (e.g., cows), possibly feral (e.g., swine), or pest species (e.g., mice and rats). Studies have documented *Cx. quinquefasciatus* feeding upon all of these mammals at low levels in urban ecosystems [42,49]. Although we detected these mammal species, the proportion of dog, mouse, and rat blood meals is likely not proportional to their abundance. Thus, one shortcoming of using iDNA to detect vertebrate biodiversity is that the behavior of the insect will dictate the species that are detected, leading to a bias and lack of correlation to real density of potential host species. Thus, we recommend that iDNA be used as a complement to traditional trapping techniques and to capture as many species of hematophagous invertebrates as possible to limit behavioral bias.

Reptiles, while not commonly detected in *Cx. quinquefasciatus* blood meal studies, can play a role in the life cycle of *Cx. quinquefasciatus*. In Puerto Rico, previous research has shown that this species will feed on reptiles in rural areas [50]. Another study in Grenada found reptiles consisted of 5% of *Cx. quinquefasciatus* blood meals in semirural neighborhoods [51]. We detected five reptile blood meals (<1%), which is below the percentage in the previously mentioned studies despite the abundance of reptiles in the SJMA [52,53]. One possible explanation for the low level of reptile blood meals is the abundance of birds, particularly chickens. If *Cx. quinquefasciatus* is ornithophilic in Puerto Rico, then access to high densities of large, slow-moving birds will distract the mosquito from other hosts. However, it could also be a function of trap sites, as some of the traps were placed in proximity to penned chickens (Yee, personal observation).

We captured only a few blood-engorged *Ae. aegypti*, but they were approximately evenly distributed among trapping sites and dates (Table 3 and Table 4). Thus, we can compare the hosts that we identified to other studies of *Ae. aegypti* host choice in Puerto Rico. A study in rural Puerto Rico identified a range of non-human vertebrates, mostly mammals, fed upon by *Ae. aegypti* [54]. Individuals that we collected in the SJMA fed upon the domestic mammal and bird species previously documented, and we identified a wild bird species, the green heron (*Butorides virescens*), in a single blood meal. Fitzpatrick et al. [51] detected blood meals from chickens and a non-chicken bird in Grenada. Other studies have demonstrated that *Ae. aegypti* will feed upon birds, usually chickens, that are associated with humans [51,54,55], but it less commonly feeds upon other bird species. Most studies have found the host range of *Ae. aegypti* to be limited, and thus, although using iDNA from this species is useful for epidemiology, it is not as useful for biodiversity surveys when compared to other less-specialized hematophagous arthropods.

### 4.4. Implications for Zoonotic Pathogen Transmission

Multiple vector-borne pathogens are present in Puerto Rico, including dengue virus (DENV), Zika virus (ZIKV), chikungunya virus (CHIKV), and West Nile virus (WNV) [56,57,58]. Given the threat to humans and animals, it is important to assess vector feeding behavior to develop appropriate risk models. We evaluated blood meals from two vector species; *Aedes aegypti* transmits CHIKV, DENV, and ZIKV, while *Cx. quinquefasciatus* transmits WNV. With the small sample sizes of *Ae. aegypti* in this study, we could not infer anything novel about the transmission potential of viruses by *Ae. aegypti* other than to confirm that in the SJMA, humans and other mammals are common hosts, with birds playing a minor role.

Of the viruses listed above, WNV is predominantly an avian virus that is typically enzootic in certain wild bird species. West Nile virus has been present in Puerto Rico with documented transmission in bird species since at least 2004 [59,60]. However, there have only been a few documented cases of infections in humans and no outbreaks [61]. There is evidence that wild bird species in Puerto Rico are competent hosts of WNV, but that transmission to humans and horses is minimal [62]. In an urban environment with densely populated neighborhoods, such as the ones sampled in this study, highly abundant vector populations could result in regular transmission to humans. We have shown that the main host of *Cx. quinquefasciatus* in San Juan neighborhoods is the domestic chicken. Chickens are ineffective WNV amplification hosts and do not readily infect mosquitoes [63]. Compared to chickens, the percentage of blood meals from amplifications host, such as certain passerines, was quite low.

The “dilution effect” hypothesis suggests that high abundances of inefficient host species can interfere with transmission networks, thus reducing the risk of vector-borne transmission [64]. This ecological mechanism has been proposed for the pathogen that causes Lyme disease, and also for WNV in areas of high bird diversity [65,66,67]. This hypothesis is controversial, with some studies failing to support it in favor of the hypothesis that higher biodiversity actually increases disease risk; however, no consensus has yet emerged [67]. In the SJMA, we can speculate that a type of dilution effect is a potential reason for low WNV transmission. The high densities of chickens, which do not transmit the virus, may attract *Cx. quinquefasciatus*, which then limits the biting of humans and other susceptible domestic animals. Further, the relatively low biodiversity in the SJMA is a limiting factor, as bird species that amplify and transmit the virus are either not as common, or the mosquitoes choose not to focus on these species. Further studies designed to specifically test the dilution effect hypothesis could help determine the strength of this mechanism in regulating WNV transmission, especially across urban landscapes where socioeconomic gradients likely affect host diversity.

## 5. Conclusions

As the human population grows, conflict between wild animals and humans will become more of a concern for human health and for the persistence of native wildlife species. When urban environments grow, biotic homogenization contributes to a loss of global biodiversity [68]. Through this process, exotic species are introduced into and around urban centers, and native species decline or are extirpated. Exotic pathogens often accompany the introduced species, which can have profound effects on human health, animal welfare, and the persistence of native biodiversity [69,70]. Using iDNA to explore the ecology of urban insects can highlight aspects of urban ecosystems that affect biodiversity and pathogen transmission pathways, and reveal ways to manage human-animal conflict and preserve functioning ecosystems. In this study, we have shown that iDNA is an effective tool to highlight feeding patterns of urban hematophagous insects to detect the presence of sympatric wildlife in different neighborhoods and understand the risk of vector-borne pathogen transmission to humans.

## Figures and Tables

**Table 1:** Socio-economic variables by neighborhood in the San Juan Metropolitan Area. Mean number of abandoned homes, parks, freshwater bodies, and litter items are mean values calculated from foot surveys (*n* = 103) in October 2018, January 2019, and May 2019. The remainder of the variables are mean values calculated from the U.S. Census data (2018; *n* = 21). The neighborhood abbreviations are: CA—Cataño, MP—Martin Peña, PN—Puerto Nuevo, RP—Río Piedras, TO—Torrecilla, VM—Vistamar, VV—Villa Venecia (Scavo et al., in revision).

Variable	CA	MP	PN	RP	TO	VM	VV
Number of abandoned homes	0.769	0.769	0.461	0.080	0.538	0.308	0.308
Number of parks	0.000	0.308	0.000	0.167	0.077	0.154	0.000
Number of freshwater bodies	0.231	0.385	0.000	0.000	0.000	0.153	0.000
Number of litter items	22.5	41.5	20.8	5.1	19.4	15.3	4.1
Human population density per mi²	1509	681	629	630	1433	1001	641
Proportion unemployment	0.180	0.447	0.306	0.332	0.586	0.293	0.167
Proportion with college education	0.227	0.164	0.386	0.687	0.204	0.519	0.614
Proportion below poverty	0.599	0.626	0.445	0.246	0.536	0.246	0.147
No health insurance	118	114	143	70	256	162	73
Median household income (USD)	10,000	10,000	10,000	15,000	10,000	25,000	65,000

**Table 2:** Molecular blood meal identifications to class and species, number of times that species was detected in an individual mosquito (*n*) and frequency of identification for two mosquito species collected in the San Juan Metropolitan Area, Puerto Rico.

Class	Species	*Culex quinquefasciatus*		*Aedes aegypti*	
		*n*	Frequency	*n*	Frequency
Aves	*Agapornis fischeri*	1	0.002		
Aves	*Butorides virescens*			1	0.056
Aves	*Cairina moschata*	1	0.002		
Aves	*Coereba flaveola*	3	0.005		
Aves	Columbidae	1	0.002		
Aves	*Columbina passerina*	1	0.002		
Aves	*Gallus gallus*	443	0.756	1	0.056
Aves	*Nyctanassa violacea*	5	0.009		
Aves	*Nycticorax nycticorax*	1	0.002		
Aves	*Nymphicus hollandicus*	1	0.002		
Aves	*Passer domesticus*	4	0.007		
Aves	*Quiscalus niger*	4	0.007		
Aves	*Turdus* sp.	1	0.002		
Aves	*Tyrannus dominicensis*	1	0.002		
Aves	*Zenaida asiatica*	4	0.007		
Aves	*Zenaida aurita*	1	0.002		
Aves	*Zenaida macroura*	1	0.002		
Aves	*Zenaida* sp.	3	0.005		
Total Aves		476		2	
Mammalia	*Bos taurus*	1	0.002		
Mammalia	*Canis lupus familiaris*	60	0.102	8	0.444
Mammalia	*Felis catus*	11	0.019	1	0.056
Mammalia	*Homo sapiens*	20	0.034	7	0.389
Mammalia	*Mus musculus*	8	0.014		
Mammalia	*Rattus rattus*	1	0.002		
Mammalia	*Sus scrofa*	4	0.007		
Total Mammalia		105		16	
Reptilia	*Iguana* sp.	5	0.009		
Total		586		18	

**Table 3:** Molecular blood meal identifications separated by trapping date. Identifications are presented for class and species. The number of times that species was detected in an individual mosquito (*n*) and frequency of identification are presented for two mosquito species collected in the San Juan Metropolitan Area, Puerto Rico.

Month/Year		*Culex quinquefasciatus*							*Aedes aegypti*					
	Total	Class	*n*	Frequency	Species	*n*	Freq	Total	Class	*n*	Frequency	Species	*n*	Freq
01/2018	31	Aves	24	0.77	*Coereba flaveola*	1	0.03							
					*Gallus gallus*	23	0.74							
		Mammalia	7	0.23	*Canis lupus familiaris*	6	0.19							
					*Homo sapiens*	1	0.03							
03/2018	67	Aves	52	0.78	*Agapornis fischeri*	1	0.01	2	Mammalia	2	1.00	*Canis lupus familiaris*	1	0.50
					*Columbidae*	1	0.01					*Homo sapiens*	1	0.50
					*Gallus gallus*	49	0.73							
					*Nyctanassa violacea*	1	0.01							
		Mammalia	15	0.22	*Canis lupus familiaris*	10	0.15							
					*Felis catus*	1	0.01							
					*Homo sapiens*	4	0.06							
05/2018	168	Aves	138	0.82	*Cairina moschata*	1	0.01	2	Mammalia	2	1.00	*Canis lupus familiaris*	2	1.00
					*Coereba flaveola*	2	0.01							
					*Columbina passerina*	1	0.01							
					*Gallus gallus*	128	0.76							
					*Nyctanassa violacea*	1	0.01							
					*Passer domesticus*	1	0.01							
					*Quiscalus niger*	3	0.02							
					*Zenaida asiatica*	1	0.01							
		Mammalia	30	0.18	*Bos taurus*	1	0.01							
					*Canis lupus familiaris*	13	0.08							
					*Felis catus*	6	0.04							
					*Homo sapiens*	4	0.02							
					*Mus musculus*	5	0.03							
					*Sus scrofa*	1	0.01							
10/2018	73	Aves	61	0.84	*Gallus gallus*	59	0.81	6	Aves	1	0.17	*Butorides virescens*	1	0.17
					*Passer domesticus*	1	0.01		Mammalia	5	0.83	*Canis lupus familiaris*	2	0.33
					*Tyrannus dominicensis*	1	0.01					*Homo sapiens*	3	0.50
		Mammalia	12	0.16	*Canis lupus familiaris*	5	0.07							
					*Felis catus*	1	0.01							
					*Homo sapiens*	4	0.05							
					*Sus scrofa*	2	0.03							
01/2019	103	Aves	90	0.87	*Gallus gallus*	87	0.84	2	Mammalia	2	1.00	*Homo sapiens*	2	1.00
					*Nyctanassa violacea*	1	0.01							
					*Passer domesticus*	1	0.01							
					*Zenaida aurita*	1	0.01							
		Mammalia	11	0.11	*Canis lupus familiaris*	11	0.11							
		Reptilia	2	0.02	*Iguana* sp.	2	0.02							
05/2019	144	Aves	111	0.77	*Gallus gallus*	97	0.67	6	Aves	1	0.17	*Canis lupus familiaris*	3	0.50
					*Nyctanassa violacea*	2	0.01		Mammalia	5	0.83	*Felis catus*	1	0.17
					*Nycticorax nycticorax*	1	0.01					*Gallus gallus*	1	0.17
					*Nymphicus hollandicus*	1	0.01					*Homo sapiens*	1	0.17
					*Passer domesticus*	1	0.01							
					*Quiscalus niger*	1	0.01							
					*Turdus sp*	1	0.01							
					*Zenaida asiatica*	3	0.02							
					*Zenaida macroura*	1	0.01							
					*Zenaida* sp.	3	0.02							
		Mammalia	30	0.21	*Canis lupus familiaris*	15	0.10							
					*Felis catus*	3	0.02							
					*Mus musculus*	3	0.02							
					*Rattus rattus*	1	0.01							
					*Sus scrofa*	1	0.01							
					*Homo sapiens*	7	0.05							
		Reptilia	3	0.02	*Iguana* sp.	3	0.02							

**Table 4:** Molecular blood meal identifications separated by trapping site. Identifications are presented for class and species. The number of times that species was detected in an individual mosquito (*n*) and frequency of identification are presented for two mosquito species collected in the San Juan Metropolitan Area, Puerto Rico.

*Culex quinquefasciatus* Blood Meal Identification											*Aedes aegypti* Blood Meal Identification				
Site	Total *n*	Class	*n*	Frequency	Species	*n*	Frequency	Site	Total *n*	Class	*n*	Frequency	Species	*n*	Frequency
C	1	Aves	1	1.000	*Coereba flaveola*	1	1.000	C	2	Mammalia	2	1.000	*Homo sapiens*	2	1.00
CA	237	Aves	195	0.823	*Coereba flaveola*	2	0.008	CA	3	Mammalia	3	1.000	*Homo sapiens*	3	1.00
					*Columbina passerina*	1	0.004								
					*Gallus gallus*	189	0.797								
					*Passer domesticus*	2	0.008								
					*Tyrannus dominicensis*	1	0.004								
		Mammalia	38	0.160	*Bos taurus*	1	0.004								
					*Canis lupus familiaris*	19	0.080								
					*Homo sapiens*	9	0.038								
					*Mus musculus*	6	0.025								
					*Sus scrofa*	3	0.013								
		Reptilia	4	0.017	*Iguana sp.*	4	0.017								
MP	32	Aves	32	1.000	*Gallus gallus*	30	0.938	MP	2	Mammalia	2	1.000	*Canis lupus familiaris*	1	0.50
					*Nycticorax nycticorax*	1	0.031						*Homo sapiens*	1	0.50
					*Turdus* sp.	1	0.031								
PN	6	Aves	3	0.500	*Columbidae*	1	0.167								
					*Gallus gallus*	1	0.167								
					*Passer domesticus*	1	0.167								
		Mammalia	3	0.500	*Canis lupus familiaris*	1	0.167								
					*Homo sapiens*	2	0.333								
RP	4	Aves	2	0.500	*Gallus gallus*	1	0.250	RP	4	Mammalia	4	1.000	*Canis lupus familiaris*	3	0.75
					*Nymphicus hollandicus*	1	0.250						*Felis catus*	1	0.25
		Mammalia	2	0.500	*Canis lupus familiaris*	2	0.500								
T	296	Aves	237	0.801	*Cairina moschata*	1	0.003	T	3	Aves	1	0.333	*Gallus gallus*	1	0.33
					*Gallus gallus*	218	0.736			Mammalia	2	0.667	*Canis lupus familiaris*	1	0.33
					*Nyctanassa violacea*	5	0.017						*Homo sapiens*	1	0.33
					*Passer domesticus*	1	0.003								
					*Quiscalus niger*	4	0.014								
					*Zenadia asiatica*	4	0.014								
					*Zenaida macroura*	1	0.003								
					*Zenaida* sp.	3	0.010								
		Mammalia	58	0.196	*Canis lupus familiaris*	34	0.115								
					*Felis catus*	11	0.037								
					*Homo sapiens*	9	0.030								
					*Mus musculus*	2	0.007								
					*Rattus rattus*	1	0.003								
					*Sus scrofa*	1	0.003								
		Reptilia	1	0.003	*Iguana* sp.	1	0.003								
VM/VV	10	Aves	6	0.600	*Agapornis fischeri*	1	0.100	V	1	Aves	1	1.000	*Butorides virescens*	1	1.00
					*Gallus gallus*	4	0.400		3	Mammalia	3	1.000	*Canis lupus familiaris*	1	1.00
					*Zenaida aurita*	1	0.100								
		Mammalia	4	0.400	*Canis lupus familiaris*	4	0.400								

**Table 5:** Diversity indices for vertebrates identified from mosquito blood meals collected at six different trapping dates in the San Juan Metropolitan Area, Puerto Rico. The indices listed are Shannon’s entropy (*H′*), Gini–Simpson (*D*), and rarefied species richness (*S_R_*).

Date	*H′*	*D*	S_R_
01/2018	0.76	0.59	4.00
03/2018	1.05	0.47	4.34
05/2018	1.06	0.53	5.94
10/2018	0.76	0.62	4.39
01/2019	0.85	0.58	3.40
05/2019	0.88	0.61	7.08

## Data Availability

DNA sequencing data were deposited in NCBI Genbank and Sequencing Read Archive.

## Supplementary Materials

The following are available online at https://www.mdpi.com/2075-4450/12/2/129/s1, Table S1: PCR Primers, Table S2: Bioinformatics code, Table S3: Tables with sequencing results for each sample.
