# Supplementary material for: Temporal and Spatial Blood Feeding Patterns of Urban Mosquitoes in the San Juan Metropolitan Area, Puerto Rico
Source: Insects. 2021 Feb 2;12(2):129. doi: 10.3390/insects12020129 (PMC7913113; doi:10.3390/insects12020129)
Supplement: Supplementary file 1 [file insects-12-00129-s001.zip › Table S2-bioinformatics.pdf]

Table S2. Code for bioinformatics processing of mitochondrial 12S rRNA gene sequences from mosquito blood meals collected in the San Juan Metropolitan Area, Puerto Rico.

Trimmomatic – Trim and filter reads based on quality

```
for R1 in *R1*; do R2=${R1//R1_001.fastq.gz/R2_001.fastq.gz};
R1paired=${R1//.fastq.gz/_paired.fastq.gz}; R1unpaired=${R1//.fastq.gz/_unpaired.fastq.gz};
R2paired=${R2//.fastq.gz/_paired.fastq.gz}; R2unpaired=${R2//.fastq.gz/_unpaired.fastq.gz}; echo "java
-jar /home/matt_hopken/software/trimmomatic/Trimmomatic-0.36/trimmomatic-0.36.jar PE -threads
32 -phred33 $R1 $R2 $R1paired $R1unpaired $R2paired $R2unpaired
ILLUMINACLIP:/home/matt_hopken/software/trimmomatic/Trimmomatic-0.36/adapters/NexteraPE-
PE_AI_primer.fa:2:30:10 CROP:250 SLIDINGWINDOW:4:20 MINLEN:75" >> trimmomatic.cmds; java -jar
/home/matt_hopken/software/trimmomatic/Trimmomatic-0.36/trimmomatic-0.36.jar PE -threads 32 -
phred33 $R1 $R2 $R1paired $R1unpaired $R2paired $R2unpaired
ILLUMINACLIP:/home/matt_hopken/software/trimmomatic/Trimmomatic-0.36/adapters/NexteraPE-
PE_AI_primer.fa:2:30:10 CROP:250 SLIDINGWINDOW:4:20 MINLEN:75; done
```

USEARCH

Merge paired reads

```
nice -n 17 bash -c ‘for R1 in *R1_001_paired.fastq; do echo $R1; R2=${R1//R1/R2};
outfile=${R1//L001_R1_001_paired.fastq/merged.fastq}; usearch -fastq_mergepairs $R1 -reverse $R2 -
fastqout $outfile; done’
```

Quality filter using expected error (vsearch because usearch is only 32 bit and can’t handle the data)

```
for file in *merged.fastq; do echo $file; outfile=${file//merged.fastq/filtered.fasta}; vsearch -fastq_filter
$file -fastq_maxee 1.0 -relabel Filt -fastaout $outfile; done
```

dereplicate (create file with uniq sequences)

```
for file in *filtered.fasta; do echo $file; outfile=${file//filtered.fasta/uniques.fasta}; usearch -
fastx_uniques $file -sizeout -relabel Uniq -threads 48 -fastaout $outfile; done
```

Cluster 97% identity OTUs

```
for file in *uniques.fasta; do echo $file; outfile=${file//uniques.fasta/otus.fasta}; usearch -cluster_otus
$file -otus $outfile -relabel Otu; done
```



```
for file in *uniques.fasta; do echo $file; outfile=${file//uniques.fasta/otus.fasta}; usearch -cluster_otus
$file -otus $outfile -relabel Otu; done
```



```
for file in *uniques.fasta; do echo $file; outfile=${file//uniques.fasta/zotus.fasta};
tab=${file//uniques.fasta/unoise3.txt}; usearch -unoise3 $file -zotus $outfile -tabbedout $tab; done
```

Annotate zotus with number of reads

```
for reads in *filtered.fasta; do echo $reads; zotus=${reads//filtered.fasta/zotus.fasta};
otutab=${reads//filtered.fasta/otutab.txt}; outfile=${zotus//append.fasta/depth.fasta};
```

size=${reads/filtered.fasta/size.txt}; usearch -otutab $reads -otus $zotus -otutabout $otutab -dbmatched $outfile -sizeout; done

Prepend file name to sequence in zotu file

for file in *zotus.fasta; do echo $file; outfile=${file//zotus.fasta/zotus_append.fasta}; awk '1;/>/{ print FILENAME}' $file > $outfile; done

for file in *zotus.fasta; do echo $file; outfile=${file//zotus.fasta/zotus_append.fasta}; awk 'BEGIN {OFS=","} {print $0,FILENAME}' $file > $outfile; done

Taxonomic assignment

Downloaded MIDORI database for srRNA (12s) uniq .fasta from the MIDORI web server.

http://www.reference-midori.info/download.php#

for file in *zotus.fasta; do echo $file; outfile=${file//fasta/hits_blast6out.txt}; usearch -usearch_global $file -db MIDORI_UNIQ_GB238_srRNA_SINTAX.fasta -id 0.9 -strand both -top_hits_only -blast6out $outfile; done

Add file name to each line in taxonomic ID file

for file in *blast6out.txt; do echo $file; outfile=${file//blast6out.txt/blast6out_append.txt}; awk 'BEGIN {OFS=","} {print $0,FILENAME}' $file > $outfile; done

BLAST search to validate usearch_global

for file in *zotus.fasta; do echo $file; outfile=${file//zotus.fasta/blast_tab.out}; blastn -db nt -query $file -max_target_seqs 1 -outfmt "6 qseqid sseqid stitle length qcovs pident mismatch evalue bitscore" -num_threads 32 -out $outfile; done

append file name to each line in BLAST results

for file in *blast_tab.out; do echo $file; outfile=${file//tab.out/append.out}; awk 'BEGIN {OFS=","} {print $0,FILENAME}' $file > $outfile; done

concatenate BLAST results

cat *blast_append.out > all_blast.out
